# Variation in Prosthetic Joint Infection and treatment strategies during 4.5 years of follow-up after primary joint arthroplasty using administrative data of 41397 patients across Australian, European and United States hospitals

**DOI:** 10.1186/s12891-017-1569-2

**Published:** 2017-05-22

**Authors:** Perla J. Marang-van de Mheen, Ellie Bragan Turner, Susan Liew, Nora Mutalima, Ton Tran, Sten Rasmussen, Rob G. H. H. Nelissen, Andrew Gordon

**Affiliations:** 1 Department of Medical Decision Making, Leiden University Medical Center, J10-S, PO Box 9600, 2300 RC Leiden, The Netherlands; 2 Dr Foster Ltd, London, UK; 3 Department of Orthopaedic Surgery, Alfred Hospital, Melbourne, Australia; 4 Department of Orthopaedic Surgery, Monash Health, Dandenong, Australia; 5 Department of Surgery, Monash University, Dandenong, Australia; 6 Orthopaedic Surgery Research Unit, Aalborg University Hospital, Aalborg, Denmark; 7 Department of Clinical Medicine, Aalborg University, Aalborg, Denmark; 8 Department of Orthopaedic Surgery, Leiden University Medical Center, Leiden, The Netherlands; 9 Department of Orthopaedic Surgery, Sheffield Teaching Hospitals NHS trust, Sheffield, UK

**Keywords:** Periprosthetic Joint Infection, International variation, Total joint replacement, Treatment strategies

## Abstract

**Background:**

To identify best practices and quality improvement initiatives, we aimed to assess whether the incidence of Periprosthetic Joint Infection (PJI) and treatment strategies differed across patients treated in Australian, European and United States (US) hospitals.

**Methods:**

Routinely collected administrative data for 41397 patients undergoing a primary total hip or knee arthroplasty between July 2007-December 2010 across 22 hospitals were included. Patients were followed for 2 years looking for PJI occurrence, defined as early (within 4 weeks) and late PJI, and surgical treatment during 2.5 years after PJI diagnosis. Logistic and Poisson regression models were used to test for differences in PJI occurrence and treatment strategies across the three geographical regions, adjusted for age, sex, joint and Elixhauser comorbidity groups.

**Results:**

PJI occurrence varied from 1.4% in European to 1.7% in Australian patients, which were significantly higher than US patients after adjustment for patient characteristics (OR 1.24 [1.01–1.52] and 1.40 [1.03–1.91] respectively). Early PJIs varied between 0.3% in European to 0.6% in Australian patients, but adjusted rates were similar. Revision following PJI was significantly lower in Australian than in US patients (﻿OR 0.46 [0.25–0.86]) as were the total number of revisions (﻿RR 0.51 [0.36–0.71]) and number of surgical procedures (﻿RR 0.60 [0.44–0.81]) used to treat PJI.

**Conclusion:**

The overall PJI rate was significantly higher in Australian patients, but fewer procedures were needed to treat these PJIs. Future research should reveal whether this reflects PJIs caught earlier or less severe when diagnosed, and whether this is associated with the longer length of stay after primary arthroplasty in Australian hospitals.

**Electronic supplementary material:**

The online version of this article (doi:10.1186/s12891-017-1569-2) contains supplementary material, which is available to authorized users.

## Background

Worldwide about two million total hip (THA) and total knee arthroplasties (TKA) are performed annually, with the majority in the United States and Europe [1, 2]. The number of arthroplasties is expected to increase considerably over the next decades, [3, 4] due to the ageing population, an increasing prevalence of obesity, and the demand and expectation of these procedures in increasingly younger patients [5]. This increased burden on our healthcare systems will have considerable societal and economic consequences [6].

Although these procedures are very effective in reducing pain and improving functionality of patients, complications do occur and have shown to vary between hospitals [7]. Periprosthetic Joint Infection (PJI) following primary THA or TKA is one of the most devastating complications, with rates typically around 1–2% and slightly higher for TKA than THA [7–10]. The occurrence of PJI has huge impact on the patient, such as an increased rate of mortality and/or readmission to hospital [11] with the resulting experience of a PJI being a major trauma for which patients would like psychological support [12]. Projections indicate a three-fold increase of PJIs by the year 2030 [3, 13]. However, PJI rates may vary, not only between hospitals but also between health care systems, and with respect to treatment strategies used. By studying these variations, we may be able to identify best practices and thus directions for quality improvement. Furthermore, with the expected growth in THA and TKA procedures to be performed in the coming decades, the rise in antimicrobial resistance of organisms and the increased complexity of patients being cared for, the number of PJIs is likely to increase accordingly along with significant costs to treat PJI [11, 14].

The aim of the present study is firstly to assess whether PJI occurrence differs across patients treated in hospitals from different geographical regions (Australia, Europe and United States (US)), and secondly to examine whether treatment strategies for PJI differ between these patient groups.

## Methods

### Patients

Patient data from the Global Comparators Project were used, in which hospitals from various countries all over the world share their experiences and compare their outcomes using routinely collected administrative admission data. As previously described, diagnoses and procedures were combined into groups and comorbidities were defined within this Project, which were matched across countries to reconcile the different coding systems being used [15]. For the present study, all patients who underwent a primary THA or TKA in the period July 2007-December 2010 were selected; data were available for patients from 22 hospitals across six countries (Australia, Belgium, Denmark, Netherlands, United Kingdom (UK), and the US). We distinguished three geographical regions: Australia (four hospitals), Europe (11 hospitals) and United States (seven hospitals). The names of individual hospitals contributing data are listed in the Additional file 1: Table A. Patients receiving a primary THA or TKA with a primary diagnosis of cancer in the same admission, that is any C-code (ICD-10) or codes between 104 and 2097 (ICD-9), were excluded.

### Definitions

All patients with a procedure code for a primary THA or TKA were followed over a 2-year period for the occurrence of a PJI during subsequent admissions. The following codes were used to define a PJI, as the primary or secondary diagnosis of an admission: 996.66 (ICD-9, for the Netherlands 996.6), T84.5 or T84.7 (ICD-10 and ICD-AM). These represent billable medical codes that can be used to indicate a diagnosis on a reimbursement claim. Only those PJIs that occurred within 2 years after the initial admission for the primary THA or TKA were included. We distinguished between early PJI (occurring within 4 weeks) and late PJI (after 4 weeks) based on clinical relevance, as treatments are likely to differ between these two groups.

The primary and secondary procedure codes of all admissions in these patients were then examined for any codes indicating a surgical treatment of the PJI, in a period of 2.5 years after PJI diagnosis. Additional file 1: Table B shows the codes used to identify the various possible treatments in the different coding systems. For the analysis, the placement of an antibiotic spacer was aggregated with revision or resection arthroplasty, as some coding systems were not able to distinguish between these groups and even more, these procedures are used in various combinations in clinical practice (i.e. the antibiotic spacer is put in when the prosthesis is removed).

In addition, the following case-mix variables were collected: age 70+ versus <70, gender and all 32 Elixhauser comorbidity groups (yes/no variable) as identified from the secondary diagnoses data.

### Statistical analysis

The mean PJI occurrence among patients treated in hospitals from the three regions with 95% confidence intervals was calculated first. This was done separately for primary THA or TKA, and separately for early and late PJI occurrence as defined above. Testing was then undertaken for differences in PJI occurrence between the three regions after adjustment for case-mix, using stepwise backwards logistic regression analysis. The following factors were included as possible confounding variables: age group, sex, Elixhauser comorbidity groups, TKA versus THA, and region. Region however, was mandatory for the model and so was not eliminated as part of the stepwise backwards analysis. An Elixhauser comorbidity group was only included if at least 10 patients with that comorbidity had also experienced a PJI, to prevent unstable estimates and improve model fit. Model fit was examined using both the C-statistic and the Nagelkerke R-squared. The same analysis was done for early PJI occurrence.

To assess whether treatment for PJI differed between patients from hospitals in different regions, we looked at the differences in the surgical procedure following the PJI. First we looked at revision following PJI using backwards logistic regression analysis within patients with a PJI diagnosis, with the same variables included as above and revision/resection (yes/no) as the dependent variable. Region was a mandatory factor in the model. However, revision surgery (i.e. one-stage revision) or resection arthroplasty surgery (i.e. removal of implant) as the first surgical procedure may indicate a more aggressive approach, compared with irrigation and debridement as the first procedure and so was subject to further sensitivity analysis with a further condition being that revision/resection (yes/no) was performed as the first surgical procedure as the dependent variable. The same independent variables as above were included and region was again a mandatory factor in the model. The same analysis was done with irrigation and debridement (yes/no) as the first surgical procedure.

But even if the first surgical procedure is more aggressive, this may still be a more effective course of action if it means that the patient undergoes fewer procedures. Therefore, the total number of surgical procedures used to treat PJI across patients from hospitals in the three regions was also examined. This was done using Poisson regression analysis, with region mandated in the model and using the same case-mix variables mentioned above. The same analysis was done for the total number of revisions/resections, to assess whether a less invasive initial approach might still result in more invasive procedures being necessary.

In all analyses, a *p*-value < 0.05 was considered to be statistically significant.

## Results

Data from 22 hospitals across the three geographical regions were included, covering 41397 primary procedures (Table 1). Patient characteristics varied between regions and in particular the percentage of patients with any comorbidities being much higher in the US. The length of stay in the US was much lower in the admission when the primary arthroplasty took place (on average 9 days shorter than Australia).

**Table 1:** Patient characteristics, PJI rate and treatment across 3 regions

	Australia	Europe	US	Total
Hospitals				
Number of hospitals	4	11	7	22
Arthroplasties				
Number of arthroplasties	3705	26993	10699	41397
% Hip	47%	50%	46%	49%
Patient characteristics at primary arthroplasty				
Average age	68.4	68.2	62.3	66.7
Median age	70	69	63	68
% Females	62%	61%	60%	61%
% with 1 or more comorbidities	42%	56%	80%	61%
Average number of comorbidities	0.8	0.9	1.7	1.1
Median number of comorbidities	0	1	2	1
Length of stay (LOS) of primary arthroplasty admission				
Average LOS in arthroplasty spell	12.4	7.7	3.3	7.0
Median LOS in arthroplasty spell	7	6	3	5
% patients in upper quartile LOS	46%	33%	2%	26%
Prosthetic joint infections (PJI)				
PJI rate within 2 years	1.7%	1.4%	1.5%	1.4%
Treatment of prosthetic joint infection within 2.5 years				
Average number of procedures	0.9	1.3	1.6	1.4
Median number of procedures	1	1	1	1

**Fig. 1:**
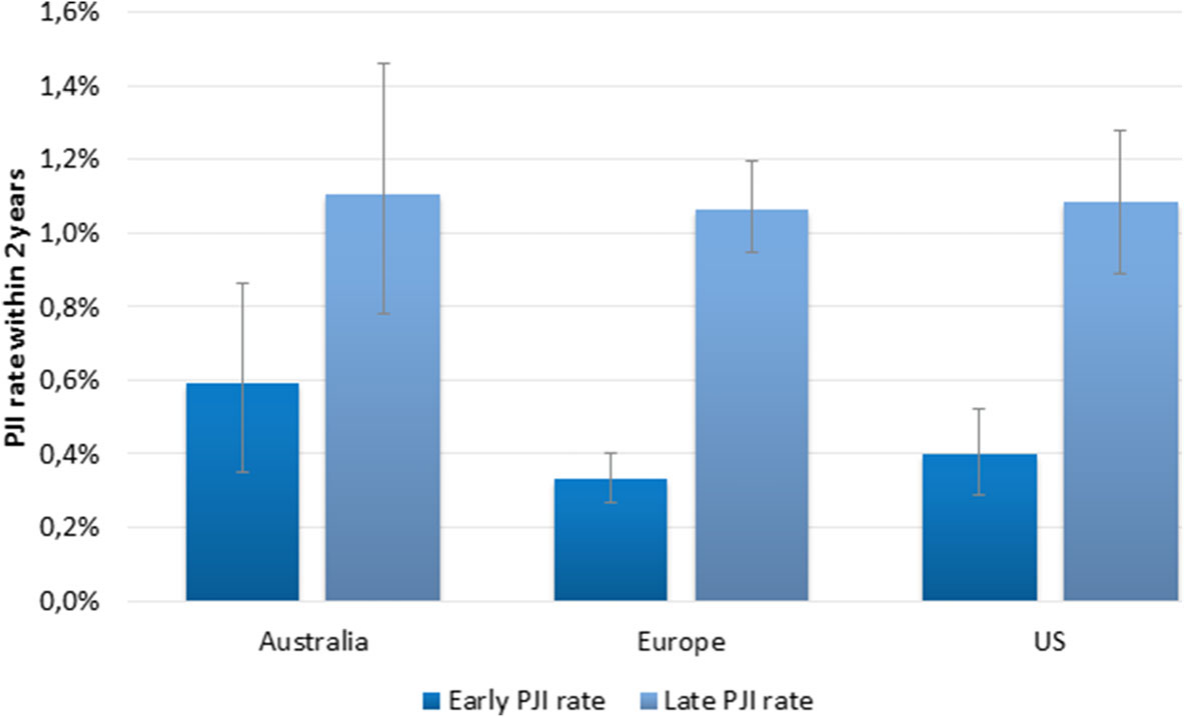
Crude early Periprosthetic Joint Infection (PJI) rate (<4 weeks) versus late PJI rate (<2 years) across 3 regions

Crude PJI rates were highest in Australian patients (1.7%) and lowest in Europe (1.4%). Figure 1 shows that the higher PJI rate in Australian patients was mainly in early PJIs occurring within 4 weeks, but that late PJI rates were similar. After adjustment for differences in patient characteristics, the likelihood of a PJI diagnosis was significantly higher in both Australian and European patients compared to the US (Table 2). Table 2 also shows that the likelihood of a PJI was significantly increased for male patients with various types of comorbidities undergoing TKA. PJI was less likely to be diagnosed in elderly patients (70+ years of age) independent from any comorbidity, which probably reflects a healthy selection of elderly patients without comorbidity undergoing THA or TKA or alternatively, these patients receive antibiotic suppression therapy to prevent PJI because they are too frail to undergo revision surgery. Looking only at early PJI, rates were similar as the US after adjustment for patient characteristics (OR 1.46 [0.86–2.47] for Australia and 0.98 [0.67–1.42] for Europe).

**Table 2:** Influence of patient characteristics and region on Periprosthetic Joint Infection (PJI) rate

Variable	Odds Ratio [95% CI]
Age > =70 years	**0.75 [0.63-0.89]**
Sex (Males versus Females)	**1.38 [1.17–1.63]**
Comorbidities	
Cardiac arrhythmias (yes/no)	1.32 [0.99–1.74]
Congestive heart failure (yes/no)	1.58 [0.98–2.53]
Rheumatoid arthritis, collagen or vascular disease (yes/no)	**1.96 [1.39–2.75]**
Diabetes uncomplicated (yes/no)	**1.28 [1.00–1.64]**
Coagulopathy (yes/no)	**2.28 [1.29–4.03]**
Fluid and electrolyte disorders (yes/no)	**1.63 [1.15–2.30]**
Depression (yes/no)	**2.04 [1.46–2.86]**
Joint (knee versus hip)	**1.52 [1.28–1.79]**
Region	
Australia versus US	**1.40 [1.03–1.91]**
Europe versus US	**1.24 [1.01–1.52]**

Significant differences are indicated in bold; Model fit: C statistic = 0,61 Nagelkerke *R* ^2^ = 0,018

**Fig. 2:**
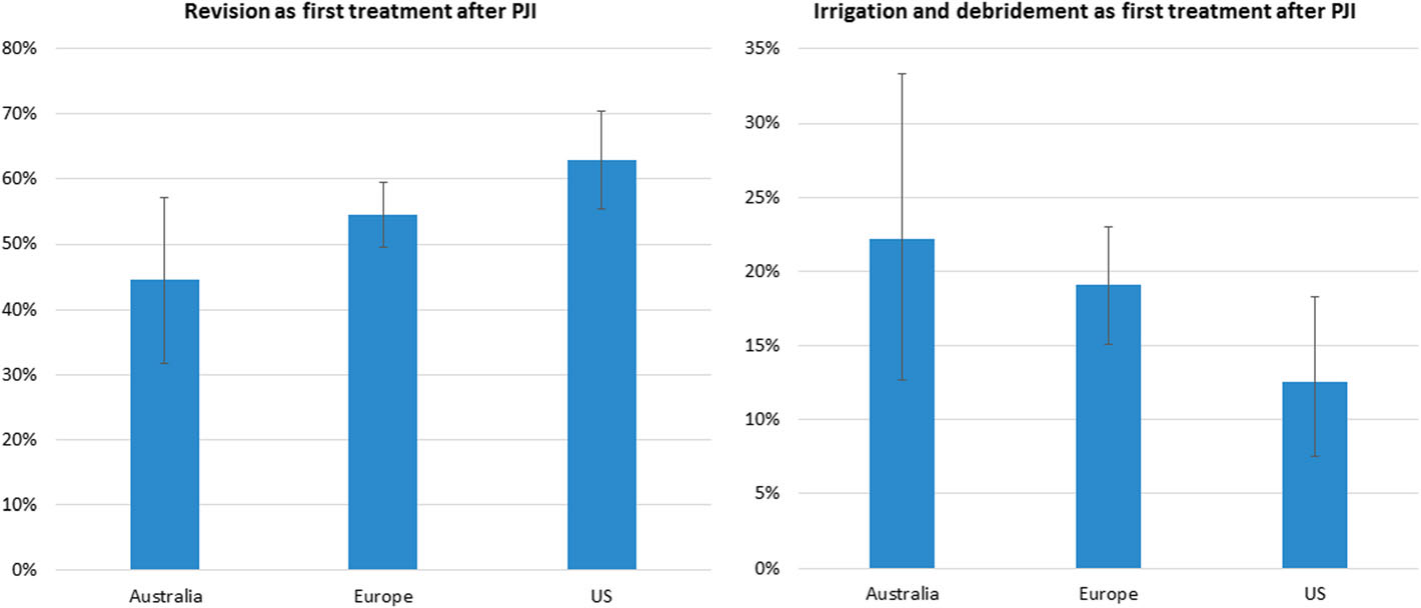
Crude rates of first treatment of Periprosthetic Joint Infection (PJI) across 3 regions

**Fig. 3:**
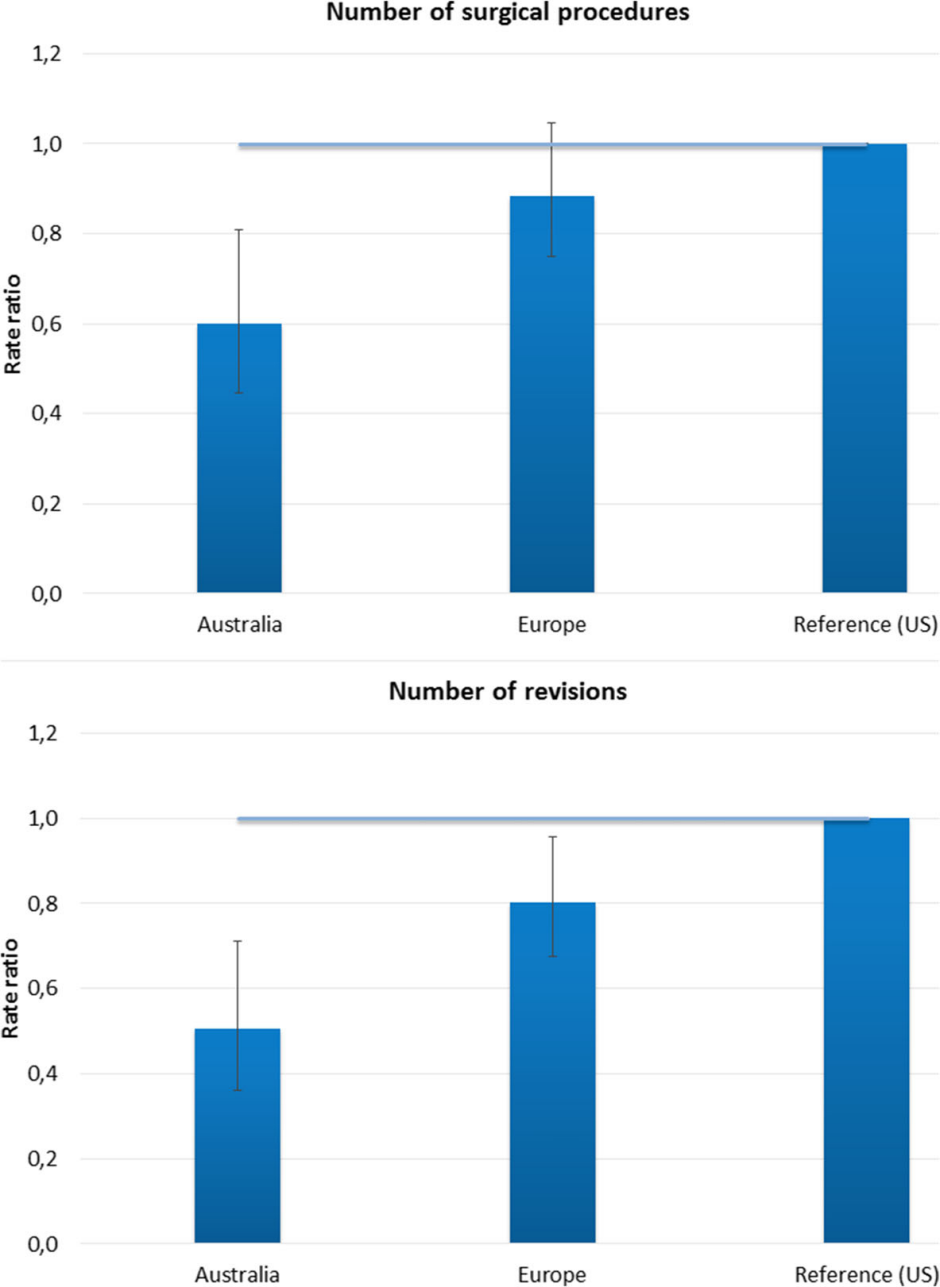
Difference between regions in number of surgical procedures and number of revisions for Periprosthetic Joint Infection (PJI), adjusted for differences in patient characteristics

Table 3 shows that after adjustment for differences in patient characteristics, chances of a revision as treatment following PJI diagnosis are significantly lower in Australian patients (54% lower). Figure 2 shows that treatment strategies seem to differ across regions with irrigation and debridement more often used as first treatment following PJI diagnosis in Australia and revision more often as first treatment in the US. After adjustment for differences in patient characteristics, chances of a revision as first treatment following PJI diagnosis was significantly lower in Australia (OR 0.50 [0.28–0.92]) and similar in Europe (OR 0.71 [0.48–1.06]) compared to the US. Differences in irrigation and debridement as first treatment were not statistically different after adjustment for patient characteristics (data not shown). Looking at the number of procedures, both the number of revisions and the total number of surgical procedures following PJI diagnosis are significantly lower in Australia after adjustment for differences in patient characteristics (Fig. 3). Europe also has significantly fewer revisions following PJI diagnosis compared to the US, but the total number of surgical procedures does not differ significantly.

**Table 3:** Influence of patient characteristics and region on having a revision as surgical treatment following Periprosthetic Joint Infection (PJI)

Variable	Odds Ratio [95% CI]
Age > =70 years	**0.62 [0.43–0.87]**
Comorbidities	
Obesity (yes/no)	0.63 [0.36–1.12]
Fluid and electrolyte disorders (yes/no)	**0.43 [0.22–0.86]**
Region	
Australia versus US	**0.46 [0.25–0.86]**
Europe versus US	0.77 [0.51–1.16]

Significant differences are indicated in bold; Model fit: C statistic = 0,62 Nagelkerke *R* ^2^ = 0,053

## Discussion

The present study has shown that PJI after primary THA and TKA is diagnosed more frequently in Australian and European patients than in US patients, but that occurrence of early PJI within 4 weeks is similar. The PJI rate was significantly higher in patients with various types of comorbidities as well as after knee arthroplasty. Treatment strategies following PJI were also different across these three regions, with revisions being less frequently used as first treatment in Australia as well as fewer revisions and a lower total number of surgical procedures. Europe also had fewer revisions following PJI diagnosis but the same total number of surgical procedures as the US. The hypothesis that would fit these findings is that PJIs in Australia are caught earlier or are less severe when diagnosed resulting in fewer surgical procedures being necessary, which should be further investigated.

Limitations of our study include the use of administratively collected data which may overestimate or underestimate the presence of comorbidities and complications, with differences across hospitals and health care systems due to different coding practices. In the present study the average number of reported comorbidities in patients was clearly higher for US patients, most likely due to the financial incentives associated with coding [16], which may result in PJI rates in the US being underestimated after adjustment for these reported comorbidities. However, reimbursement of health services in Australia also depends on clinical coding [17], but did not result in a higher number of reported comorbidities, so that it does not seem likely that this will explain the results entirely. Another issue is the ability to correctly identify PJI from administrative data. Lange et al have recently shown an 85% positive predictive value for periprosthetic hip joint infection using administrative discharge registers from Denmark, which slightly increased to 86% when combined with an infection-specific surgical procedure code [18]. Bozic et al also argued that diagnoses like PJI are less influenced by coding practices and found 99% agreement after medical record validation [7]. Related to this, variation in the definition of PJI across centres could have influenced our findings. However, given the use of clinical admission data with ICD9 or ICD10 coding, only those PJIs serious enough to warrant readmission will have been included, and less subject to variation in applied definitions. Finally, bias in procedures to treat PJI is far less likely but nevertheless there was still variation in surgical treatment strategy amongst the three health care systems, with fewer procedures required to treat PJI in Australia. When checking all the data for incomplete follow-up time after PJI diagnosis (i.e. less than 2.5 years), it was found that 4% of the PJIs in Europe had incomplete follow-up, 0% in the US and 0% in Australia. As these percentages are very low, it is not likely to affect the results to a great extent. If anything, this means that the number of revisions following PJI might have been underestimated in Europe, so that the difference with the US may be smaller in reality, but it cannot explain the difference between Australia and the US. The number of primary arthroplasties per patient was also checked, to investigate the possibility that another “primary arthroplasty” may have been coded for when in reality a revision is performed. However, numbers of more than two primary arthroplasties in a patient were extremely low, particularly in the US (0.04%, versus 0.05% in Australia and 0.07% in Europe) so this also is not likely to have influenced our results. Finally we checked the extent to which PJIs were diagnosed in the same admission as the primary arthroplasty, whereas only PJIs in subsequent admissions were counted in the primary analysis. As expected, rates were low (0.5% for Australia, 0.3% for Europe and 0.1% for US) so that the difference in PJI between patients from different regions might even have been underestimated.

It is important to note that the study findings are only based on a limited number of hospitals within the countries and regions examined. As each centre was a large academic medical unit, outcomes between centres included in our study will be fairly comparable with respect to their patient population, but may differ from other units in the selected countries. Therefore it is possible that observed differences in this study are only found among complex patients typically treated in academic centres but are not representative for patients treated in other hospitals in these regions. Still, the overall PJI rate of 1.4% within 2 years after the primary arthroplasty and 0.4% of early PJIs within 4 weeks fits well in the reported range of PJI in previous studies, when differences in duration of follow-up between studies are taken into account [7, 8, 10]. Another reason for discrepancies with previously reported rates is that some studies only include PJIs with a related procedure [7, 8]. Excluding PJI with an unrelated procedure in the present study, would result in similar PJI rates around 1% as reported in those studies. Furthermore, the factors identified to increase the likelihood of a PJI such as coagulopathy and rheumatoid arthritis as well as being higher after primary TKA, are consistent with previous reports [9, 19, 20].

## Conclusions and implications

What the present study has shown for the first time is that both PJI diagnosis and the associated treatment strategy seem to differ across patients treated in different health care systems. Discussions among collaborating hospitals did not suggest any difference in preoperative infection prophylaxis strategy but no patient level data were available to substantiate this. Combining the significantly higher PJI rate in Australia with the less aggressive treatment strategy of revisions being less often the first treatment following PJI and fewer surgical procedures, results in the hypothesis of less severe PJIs when diagnosed or PJIs caught earlier. This may be associated with the considerable longer length of stay in Australian hospitals, which may indicate (early signs of) the PJI or alternatively, being more of a ‘standard duration’ in this health care system but thereby enabling identification of the PJI compared with US hospitals discharging patients early so early PJIs may not be identified. This is supported by the difference in PJI rates diagnosed in the same admission as the primary arthroplasty. Diagnosing the PJI earlier is also likely to affect the treatment strategy, that is requiring either less aggressive or fewer procedures to be treated adequately. Another explanation may be an active surveillance system being present as for instance the Victorian Healthcare Associated Infection Surveillance System (VICNISS) reporting a significant reduction of surgical site infections over time including a reduction in deep surgical site infections [21]. However, similar surveillance systems are also present in the United States and in Europe (e.g. the CDC and the ECDC), so that it does not seem likely that this would explain the observed difference between regions. Finally, the higher number of revisions and surgical procedures being performed in the US may be due to the time of presentation or the patients’ clinical status at the time of presentation, but another thing to consider is that it may not just be clinical practice but also an effect of remuneration for procedures across the different health care systems, given for instance the higher fees for hip replacements in the US than in Australia and European countries [22].

An important question is whether the initial longer length of stay for THA and TKA in Australia, in fact is a cost-effective approach if it results in complications like PJI being caught earlier and fewer readmissions and treatments being required. Multiplying the initial longer length of stay in Australia compared to the US from Table 1, with the bed day costs of about £400 [23] and divided by the difference in number of revisions, this would amount to £410.241 health care costs per prevented revision in Australia. For Europe, health care costs would be £363.866 higher. These costs are likely to be overestimated as only hospital bed days were included in this study and patients in the US routinely go to rehabilitation centres after leaving the hospital. Assuming the same total length of stay in the US as in Europe, would still amount to £213.366 per prevented revision in Australia. These costs exceed the average costs for revision surgery of £25.974. [23, 24] Including societal costs by assuming that 50% of patients would be absent from work for 8 weeks because of the revision, amounting to £1.400 per week, costs in Australia and Europe were still considerably (£378.667 and £332.292 respectively) higher than the costs for revision surgery. Assuming a threshold of £30.000 per Quality Adjusted Life Years (QALY) for a cost-effective intervention, this would mean that 12,6 QALY’s have to be gained by preventing revisions, for the Australian strategy to be cost-effective compared to the US (11,1 QALY’s for Europe). This is highly unlikely, given that even under assumptions of temporary health loss of 50% during 6 months and 20% permanent health loss for 10% of the patients, this would only amount to about 1 QALY gained. Our results thus have important implications for future directions in clinical practice, but also strongly illustrate the value of international collaboratives allowing us to compare outcomes, learn from each other, and enable improvements in quality of care.

## Additional file

Additional file 1: Table A. Individual hospitals contributing data by region. **Table B.** Included secondary procedure codes to identify surgical treatment for PJI. (DOCX 16 kb)
