# Supplementary material for: Variation in Prosthetic Joint Infection and treatment strategies during 4.5 years of follow-up after primary joint arthroplasty using administrative data of 41397 patients across Australian, European and United States hospitals
Source: BMC Musculoskelet Disord. 2017 May 22;18:207. doi: 10.1186/s12891-017-1569-2 (PMC5441102; doi:10.1186/s12891-017-1569-2)
Supplement: Additional file 1: Table A. — Individual hospitals contributing data by region. Table B. Included secondary procedure codes to identify surgical treatment for PJI. (DOCX 16 kb) [file 12891_2017_1569_MOESM1_ESM.docx]

Additional file 1

**Table A Individual hospitals contributing data by region**

| **Region** | **Country** | **Name of hospital** |
| --- | --- | --- |
| Australia (4 hospitals) | Australia | Alfred Health |
|  | Australia | Austin Health |
|  | Australia | Melbourne Health |
|  | Australia | Monash Health |
| Europe (11 hospitals) | Belgium | UZ Leuven |
|  | Denmark | Aalborg UH |
|  | England | Cambridge Univ |
|  | England | Coventry & Warwickshire |
|  | England | Guys' & St Thomas's |
|  | England | Imperial College |
|  | England | Royal Berkshire |
|  | England | Royal United Bath |
|  | England | Sheffield |
|  | England | University College London |
|  | Holland | Leiden UMC ° |
| United States (7 hospitals) | United States | Barnes-Jewish |
|  | United States | Huntsville |
|  | United States | UC San Diego |
|  | United States | UC San Francisco |
|  | United States | UPenn |
|  | United States | UT Southwestern |
|  | United States | Yale-New Haven |

**Table B Included secondary procedure codes to identify surgical treatment for PJI**

| **Treatment** | **Code type** | **Included codes** |
| --- | --- | --- |
| Irrigation & debridement or bearing exchange | ACHI Procedures (Australia) | 4930300, 4950001 |
|  | CVV procedures (Netherlands) | 58148, 58153, 58167, 580086, 580087, 580096, 580097, 580516, 580517, 580526, 580527, 580596, 580597 |
|  | ICD-9 procedures (US, Belgium) | 0073, 0084, 8015, 8016, 8075, 8076 |
|  | OPCS 4.5 (UK) | W691, W692, W693, W801, W802, W803, W813, W814, W815 |
|  | NCSP (Denmark) | KNFS19, KNFS29, KNFS49, KNFS59, KNFS99, KNFW49, KNFW59, KNFW69, KNFW79, KNFW89, KNFW99, KNGG09, KNGS09, KNGS19, KNGS29, KNGS39, KNGS49, KNGS59, KNGS99, KNGW49, KNGW59, KNGW69, KNGW79, KNGW89, KNGW99, |
| Antibiotic spacer | ACHI Procedures (Australia) |  |
|  | CVV procedures (Netherlands) | 578055, 578056, 578057, 580016, 580017 |
|  | ICD-9 procedures (US, Belgium) | 8456, 8457 |
|  | OPCS 4.5 (UK) | W817 |
|  | NCSP (Denmark) | KNFC59, KNFG19, KNFG29, KNGG19, KNGG29, KNGC59A |
| Revision / resection arthroplasty | ACHI Procedures (Australia) | 4931200, 4932400, 4932700, 4933000, 4933900, 4934200, 4934500, 4934600, 4951500, 4952700 |
|  | CVV procedures (Netherlands) | 58146, 581460, 581461, 581462, 581463, 581464, 581469, 58147, 581470, 581471, 581472, 581473, 581474, 581475, 581479, 58151, 581510, 581511, 581512, 581513, 581514, 581515, 58152, 581520, 581521, 581522, 581523, 581524, 581525, 58165, 581650, 581651, 581652 |
|  | ICD-9 procedures (US, Belgium) | 0070, 0071, 0072, 0080, 0081, 0082, 0083, 8005, 8006, 8153, 8155 |
|  | OPCS 4.5 (UK) | W370, W372, W373, W374, W380, W382, W383, W384, W390, W392, W393, W394, W395, W400, W402, W403, W404, W410, W412, W413, W414, W420, W422, W423, W424, W425, W570, W572, W573, W574, W580, W582, W930, W932, W933, W940, W942, W943, W950, W952, W953, W954 |
|  | NCSP (Denmark) | KNFC, KNFC0, KNFC01, KNFC02, KNFC03, KNFC09, KNFC1, KNFC11, KNFC12, KNFC13, KNFC19, KNFC2, KNFC20, KNFC21, KNFC22, KNFC23, KNFC29, KNFC3, KNFC30, KNFC31, KNFC32, KNFC33, KNFC39, KNFC4, KNFC40, KNFC41, KNFC42, KNFC43, KNFC49, KNFC99, KNFU01, KNFU02, KNFU09, KNFU10, KNFU11, KNFU12, KNFU19, KNFG09, KNGC01, KNGC02, KNGC02A, KNGC03, KNGC04, KNGC09, KNGC11, KNGC12, KNGC12A, KNGC13, KNGC14, KNGC19, KNGC20, KNGC21, KNGC22, KNGC22A, KNGC23, KNGC24, KNGC29, KNGC30, KNGC31, KNGC32, KNGC32A, KNGC33, KNGC34, KNGC39, KNGC40, KNGC41, KNGC42, KNGC42A, KNGC43, KNGC44, KNGC49, KNGC59, KNGC99, KNGC99A, KNGU00, KNGU01, KNGU02, KNGU03, KNGU04, KNGU09, KNGU10, KNGU11, KNGU12, KNGU13, KNGU14, KNGU19 |
| Arthrodesis | ACHI Procedures (Australia) | 4930600, 4950901 |
|  | CVV procedures (Netherlands) | 58127, 58128, 581280, 581281, 581282 |
|  | ICD-9 procedures (US, Belgium) | 8121, 8122 |
|  | OPCS 4.5 (UK) | W601, W611, W621, W622 |
|  | NCSP (Denmark) | KNFG39, KNFG49, KNFG59, KNGG39, KNGG49, KNGG59, KNGG99 |
| Amputation | ACHI Procedures (Australia) | 4436700, 4437000, 4437300 |
|  | CVV procedures (Netherlands) | 58480, 58490 |
|  | ICD-9 procedures (US, Belgium) | 8410, 8416, 8417, 8418 |
|  | OPCS 4.5 (UK) | X091, X092, X093 |
|  | NCSP (Denmark) | KNEQ19, KNFQ09, KNFQ19, KNGQ09 |
